# A review for nutritional value of *Rumex* plants and their application in livestock and poultry

**DOI:** 10.3389/fvets.2026.1859713

**Published:** 2026-07-14

**Authors:** Miao Ma, Xinyao Li, Zengyang He, Hao Ling, Jingrui Hu, Li Zhou, Jinqiang Huang, Peng Huang, Qian Lin, Kaijun Wang, Jianguo Zeng

**Affiliations:** 1 College of Animal Science and Veterinary Medicine, Shenyang Agricultural University, Shenyang, China; 2 Chinese Medicinal Materials Breeding Innovation Center of Yuelushan Laboratory, Changsha, China; 3 Hunan Jiuanhe Biotechnology Co., Ltd., Changsha, China; 4 College of Veterinary Medicine, Hunan Agricultural University, Changsha, China; 5 Institute of Bast Fiber Crops, Chinese Academy of Agricultural Sciences, Changsha, China; 6 Hunan Provincial Key Laboratory of the Traditional Chinese Medicine Agricultural Biogenomics, Changsha Medical University, Changsha, China

**Keywords:** Rumex plants, protein feed, anti-nutritional factors, livestock, poultry

## Abstract

The development of unconventional feed resources has become a strategic imperative for the sustainable advancement of animal husbandry, particularly considering China’s increasing scarcity of high-quality protein feed supplies. Perennial herbs from the *Polygonaceae* family, particularly those in the genus *Rumex*, have attracted considerable interest. Specifically, two high-protein cultivars, Edible Grass (*Rumex patientia* L. × *Rumex tianschanicus* A. LOS, abbreviated as *R. P. × R. T.*) and *Rumex* K-1—have shown promise due to their high crude protein (CP) content (20–30%), abundance of bioactive components, and strong resistance to environmental stressors. However, anti-nutritional factors (ANFs) such as oxalic acid, phytic acid, and non-starch polysaccharides (NSP) limit the feeding value of these plants. Based on a comprehensive literature search in PubMed, Web of Science, Google Scholar, CNKI, and Scopus (2000–2025), this work methodically reviews the distribution of germplasm resources, nutritional traits—including conventional nutrients, amino acid (AA) composition, and bioactive components—as well as significant anti-nutritional aspects of *Rumex* plants. This summary provides a comprehensive overview of studies investigating the use of *Rumex* plants in livestock production, specifically for pigs, chickens, and ruminants. The findings indicate that incorporating 5 to 12% fermented *Rumex* into ruminant diets can enhance growth and meat quality. An optimal inclusion level of 3 to 10% has been shown to improve intestinal health, growth performance, and antioxidant capacity in diets for pigs and poultry. Conversely, higher inclusion levels, such as 12%, may lead to decreased production performance, highlighting the dose-dependent effects of anti-nutritional components. Current research in this area faces several obstacles, including a lack of quantified energy levels, significant variability in nutrient composition across different varieties and growth stages, and uncertain mechanisms of action for bioactive components. To support the scientific utilization and industrial development of *Rumex* plants as high-quality protein feed resources, future work should focus on variety improvement, standardization of processing technologies, fundamental studies on energy metabolism, and mechanistic investigations.

## 1 Introduction

Forecasts indicate that by 2050, the global population is expected to exceed 9.7 billion, representing a 25% increase from the current 7.8 billion. As the population continues to grow over the coming decades, the demand for premium food sources, such as beef, eggs, and milk, is anticipated to rise sharply (1). Feed serves as the primary source of animal-derived protein, with protein sources in feed categorized into edible (primarily grains, including cereals and legumes) and inedible (such as grass) components. Excessive reliance on grains to produce animal protein may exacerbate regional and global food security challenges. Consequently, the worldwide feed supply is projected to increase by 21% on a dry matter (DM) basis (equating to 1.3 billion tons) by 2025, assuming feed efficiency remains constant (2). Nevertheless, protein feed continues to be a critical limiting factor in feed formulation, with most high-quality plant protein sources presently derived from byproducts of oilseed processing, including soybean meal, cottonseed meal, and rapeseed meal.

**Figure 1:**
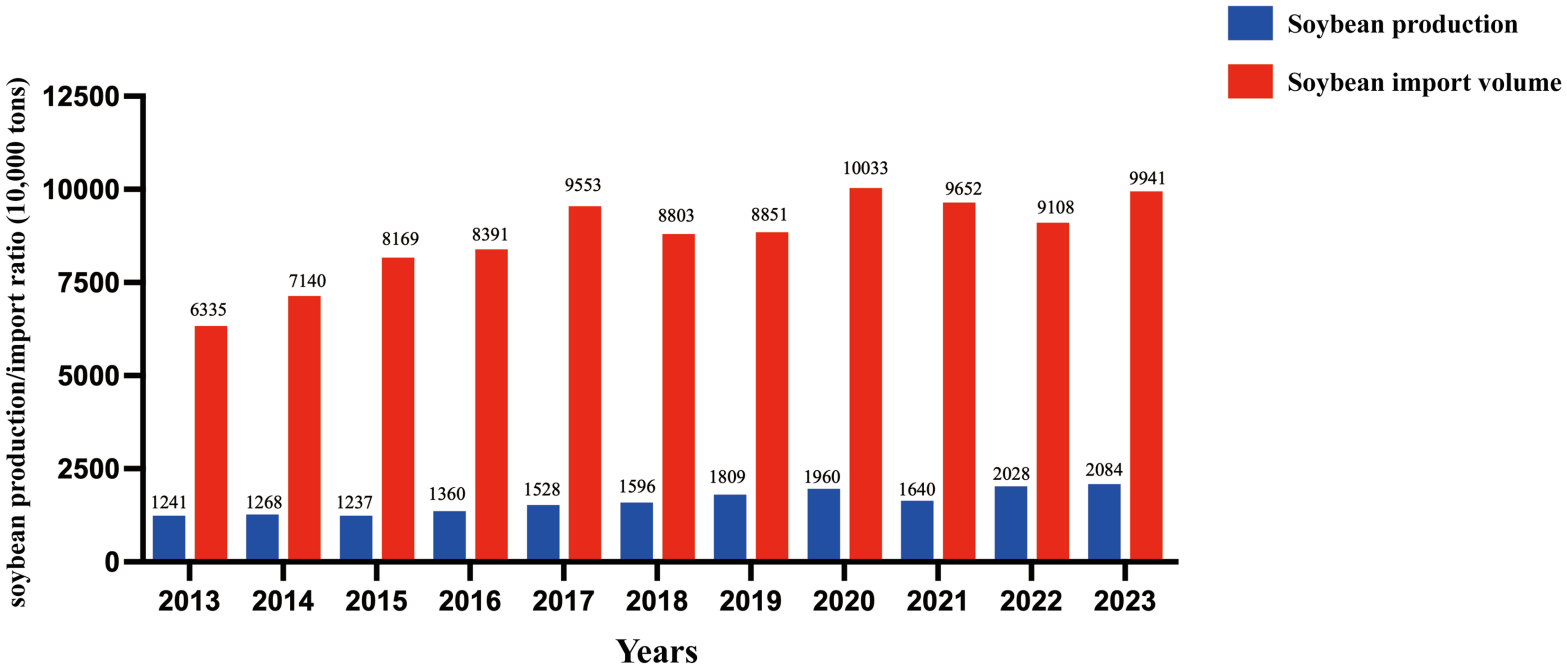
Soybean production and import volume from 2013 to 2023 in China.

China exhibits a low rate of self-sufficiency in edible protein and a high dependency on imports, primarily due to a scarcity of protein feed resources, which poses significant risks to the country’s food security. Recent data indicates that China’s edible protein self-sufficiency rate stands at 73.1%, while the protein feed self-sufficiency rate is approximately 52.6% (3). This reliance on imports raises several concerns regarding food safety and agricultural productivity. Notably, China imports a substantial quantity of soybeans; the Ministry of Agriculture and Rural Affairs’ Animal Husbandry and Veterinary Bureau reported that over 100 million tons of soybeans were imported in 2020, predominantly from Brazil, the United States, and Argentina. We have further compiled data on China’s soybean import volume and planted and harvested yields from 2013 to 2023 (Figure 1). Based on China’s soybean yield and planting area data for 2023, it is estimated that to satisfy its current soybean demand, China would require an additional 859 million mu (approximately 57.27 million hectares) of soybean cultivation area, which would account for nearly half of the nation’s total arable land (1.929 billion mu or 128.6 million hectares), thereby directly jeopardizing the country’s food security.

**Figure 2:**
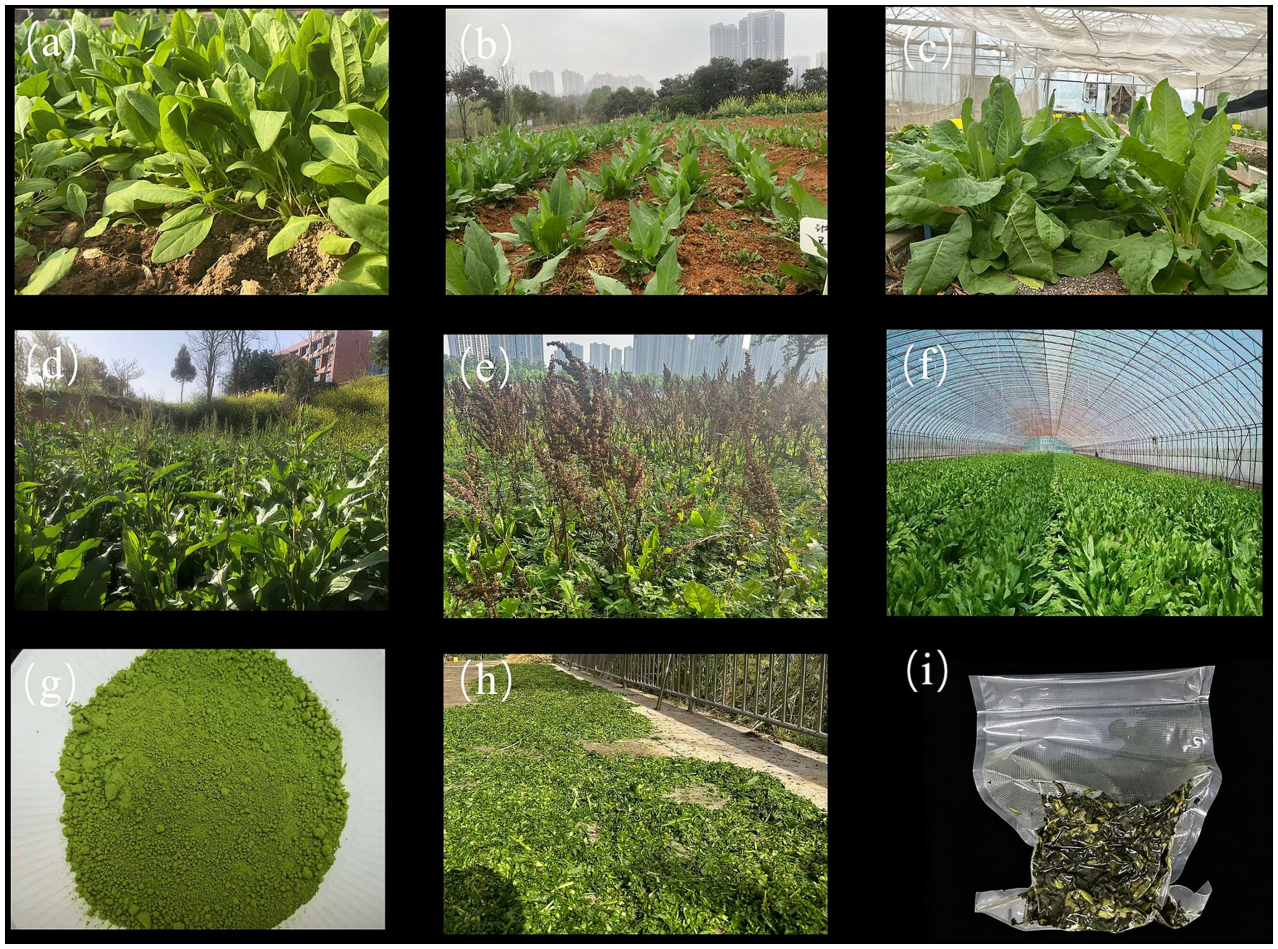
Photos of Edible Grass and Its processed into livestock feed. **(a)** EG at the seedling stage, **(b)** EG at the early vegetative growth stage, **(c)** EG at the late vegetative growth stage, **(d)** EG at the early seed-setting stage, **(e)** EG at the late seed-setting stage, **(f)** greenhouse-grown EG, **(g)** dried superfine EG powder, **(h)** wilting EG, **(i)** bagged EG silage.

In light of this, relevant practitioners have expressed considerable interest in high-protein *Rumex* plants. For instance, reports indicate that the CP concentrations of Edible Grass (*R. P* × *R. T*.) (4), *Rumex* K-1 (5), and *Rumex* OK2 (6) range from 20 to 30%, which exceeds that of alfalfa, often referred to as the ‘king of forages’ (7). These *Rumex* varieties exhibit significant potential for widespread application in agricultural production and food processing due to their high yield, robust adaptability, and elevated protein content, enabling them to maintain stable productivity and superior nutritional quality across diverse growing conditions. In recent years, feed developers and animal producers have shown substantial interest in high-protein hybrid *Rumex* species, such as Edible Grass (*R. P* × *R. T*.)(Figure 2). Their elevated CP content, multi-harvest potential, and broad adaptability position them as a promising alternative to soybean meal. To provide a comprehensive reference for the scientific utilization of this resource, this study evaluates the nutritional qualities, ANFs, and advancements in the application of *Rumex* plants in cattle and poultry production.

## 2 Overview of *Rumex* plant resources

### 2.1 Main species and distribution

The genus *Rumex* is a significant and diverse member of the *Polygonaceae* family, playing a crucial role in agricultural systems, medical practices, and global ecosystems (8). Approximately 200 species of *Rumex* are distributed worldwide, thriving in both temperate and tropical climates. Most species are herbaceous plants commonly found in roadsides, wetlands, and grasslands; some are prevalent weeds, while others have been traditionally utilized for their edible and medicinal properties (9). Although Li et al. (10) have conducted a comprehensive examination of the pharmacological characteristics, traditional applications, and geographic distribution of 35 *Rumex* species, highlighting their therapeutic potential, there remains a notable absence of a systematic compilation of germplasm resources in China and its neighboring regions, particularly concerning high-protein varieties suitable for replacing soybean meal. This research aims to provide essential genetic resource information for the breeding of new high-quality, high-protein variants by conducting a thorough assessment of *Rumex* species in this area, as documented in the Flora of China (8).

*Rumex* plants are distributed globally in temperate and subtropical climates. Notable species include *R. japonicus* Houtt and *R. patientia*, which are prevalent in Europe, Asia, and North Africa and exhibit cold tolerance (11). Additionally, *R. nepalensis* is primarily found in the Himalayan region, having adapted to high-altitude conditions, while *R. crispus* is widely dispersed throughout the Northern Hemisphere and has been extensively used in traditional medicine (12). These species possess edible, medicinal, and forage value, playing significant roles in their respective ecosystems (13). In China, *Rumex* K-1 and Edible Grass (*R. P* × *R. T*.) are currently the most widely cultivated varieties, primarily used as feed and food.

### 2.2 Edible/forage history and traditional uses

There are over 30 species of *Rumex* plants in China, distributed across both the north and south. Among them, *R. acetosa* L. is used medicinally for its entire plant, known for its effects of cooling blood and detoxifying; its tender stems and leaves are also used as vegetables and forage (Flora of China). In China, various *Rumex* species are traditionally used as folk medicinal plants. Species such as *R. japonicus* Houtt., *R. acetosa* L., *R. obtusifolius* L., *R. patientia* L., and *R. crispus* L. are widely used as a herbal medicine known as “Tu Da Huang” (Rheum root). These plants are valued for their properties of clearing heat, detoxifying, promoting blood circulation to stop bleeding, and relieving itching, and they are used to treat conditions such as scabies and various bleeding disorders (14). *Rumex* K-1 is an introduced forage variety in China. It was developed between 1974 and 1979 by the New Crop Department of the Central Botanical Garden of the Academy of Sciences of Ukraine as one of the earliest high-yielding perennial crops, widely used for forage. *Rumex* K-1 is a hybrid of two Polygonaceae species, with the maternal parent being *R. patientia* L. and the paternal parent being *R. tianschanicus* A. Los. (15). Although some reports indicate that Edible Grass (*R. P* × *R. T*.) was selected from *Rumex* K-1, according to available information from the official records of granted plant variety rights, there is currently no verifiable record confirming that this variety has been approved.

## 3 Nutritional value of *Rumex* plants

### 3.1 Conventional nutrients

Certain high-protein *Rumex* species can achieve a CP concentration of up to 40%, comparable to that of widely utilized soybean meal, as indicated in the literature (16). Consequently, high-protein *Rumex* cultivars have garnered significant interest. Notably, *Rumex* exhibits a higher CP content than other forage cultivars, such as mulberry (*Morus alba*) (17), paper mulberry (*Broussonetia papyrifera*) (18), and ramie (*Boehmeria nivea*) (19). However, energy consumption efficiency is another critical factor that restricts the application of ingredients in feed formulations. Therefore, it is not scientifically valid to replace different constituents in the feed industry solely based on CP content (20, 21). While the poultry metabolizable energy (22, 23) and swine metabolizable energy (24) values of the diets have been reported in most studies, it is important to note that these data are predominantly based on calculated rather than measured values. Different animal species exhibit varying energy values for the same dietary components, and only specific animal trials can accurately determine the true accessible energy value. The source of these energy values remains unclear in much of the existing research, presenting a significant barrier to the widespread adoption of these feed additives. Moreover, the net energy system is increasingly replacing digestible and metabolizable energy as a more precise standard for energy assessment, driven by the pursuit of precision nutrition. Consequently, the implications of fundamental energy research on livestock and poultry production warrant greater attention from feed ingredient developers.

According to Table 1, the CP content of Edible Grass(*R. P* × *R. T*.) ranges from 25 to 30%, which is approximately 70% of the protein content found in soybean meal (43% CP). However, its energy value is only equivalent to 47% of that of soybean meal (43% CP) when considering metabolizable energy (22). The digestible energy of *Rumex* K-1, at a 3% inclusion level, was determined through back-calculation based on feed formulation to be 14.27 MJ/kg, comparable to that of maize. This finding resulted in a linear decline in body weight during swine trials, potentially contributing to its suboptimal performance (24). In a low-protein diet formulation for finishing pigs that included Edible Grass (*R. P* × *R. T*.) at inclusion levels of 3.5, 5, and 6.5%, the digestible energy was measured at 9.73 MJ/kg. Pigs fed this diet demonstrated superior growth performance compared to the corn-soybean meal control group by supplementing industrial amino acids to maintain consistent levels of Lys, Met+Cys, Thr, and Trp while preserving similar amounts of NDF. These experimental results further illustrate the impact of energy value on livestock and poultry, particularly as the inclusion level of such unconventional feed ingredients increases (25).

**Table 1:** Chemical composition of corn, soybean meal and *Rumex* plants.

Species	State	DM, %	CP, %	EE, %	CF, %	Ash, %	NFE, %	Ca, %	P, %	NDF, %	ADF, %	ME, MJ/kg	DE, MJ/kg	Ref.
Corn	Air-dried	86.00	8.70	3.60	1.60	1.40	70.70	0.02	0.27	9.30	2.70	13.56	14.27	(37)
Soybean meal	Air-dried	89.00	44.00	1.90	5.20	6.10	31.80	0.33	0.62	13.60	9.60	9.83	14.26	(37)
Alfalfa	Air-dried	87.00	19.10	2.30	22.70	7.60	35.3	1.40	0.51	36.70	25.00	4.06	6.95	(37, 69)
	Air-dried	92.80	21.80	1.93	—	—	—	—	—	47.40	28.90	10.81	8.91	(27)
Edible Grass (*R. P. × R. T.*)	Air-dried	89.50	29.97	3.00	18.00	11.62	26.91	0.7	0.39	—	—	4.53	—	(22)
	Air-dried	89.85	30.15	3.17	20.82	13.78	21.93	0.29	0.46	—	—	—	—	(39)
	Air-dried	—	28.59	0.50	17.86	—	—	1.25	0.5	—	—	4.35*	—	(5)
	Air-dried	—	25.15	0.77	16.12	11.26	—	1.07	0.25	—	—	—	—	(62)
	Air-dried	—	22.48	3.80	15.80	6.30	—	1.00	0.38	27.97	17.19	—	9.73*	(25)
	Fresh	8.14	19.77	3.04	—	—	—	—	—	57.66	30.55	—	—	(55)
*Rumex* K-1	Air-dried	—	19.24	2.02	—	—	—	1.07	0.36	38.91	29.58	—	14.27*	(24)
	Fresh	7.00	24.80	4.40	—	—	—	—	—	—	—	—	—	(16)
*Rumex OK2*	Air-dried	100.00	24.00	1.75	23.30	11.90	39.90	—	—	32.70	28.20	—	—	(6)
*R. obtusifolius*	Air-dried	100.0	19.70	-	21.40	12.70	42.90	—	—	—	—	—	—	(6)
*R. acetosa* L.	Air-dried	90.39	12.19	2.26	—	10.11	—	0.34	0.22	52.59	32.55	10.11	8.34	(27)
	DM	100.00	25.00	2.50	12.90	10.00	50.00	1.07	0.44	—	—	—	—	(70)
*R. nervosus*	Air-dried	94.33	13.63	1.54	8.24	18.01	52.91	—	—	20.21	15.48	—	—	(71)
*R. nepalensis*	DM	100	22.46	3.28	19.70	5.84	48.72	0.86	0.47	—	—	—	—	(13)
	Air-dried	96.50	13.95	17.54	15.38	8.11	41.52	—	—	—	—	—	—	(72)
*R. dentatus L.*	Air-dried	95.95	13.75	12.50	9.03	8.65	52.05	—	—	—	—	—	—	(72)
*R. hastatus L.*	Air-dried	77.44	14.00	5.84	14.57	18.58	24.50	—	—	—	—	—	—	(72)
*R. acetosella L.*	—	—	17.60	—	26.42	22.32	—	0.391	1.614	—	—	—	—	(73)
*R. scutatus L.*	—	—	8.66	—	29.84	8.02	—	0.267	0.327	—	—	—	—	(73)

1. The nutritional composition of the samples was determined based on the analysis of dry matter (DM), crude protein (CP), ether extract (EE), crude fiber (CF), crude ash (Ash), and nitrogen-free extract (NFE), Neutral Detergent Fiber (NDF), Acid Detergent Fiber (ADF), along with the calculation of digestible energy (DE) and metabolizable energy (ME). 2. On a DM basis, the nitrogen-free extract (NFE) was calculated as follows: NFE (%) = 100 – (CP % + EE % + CF % + Ash %). 3. *Indicates that the metabolizable energy or digestible energy of this ingredient is back-calculated based on the diet formulation according to the “Chicken Feeding Standard” (NY/T 33–2004) and the “Pig Feeding Standard” (NY/T 65–2004), provided that there is no negative impact on the growth performance of livestock and poultry. This value may differ from the metabolizable energy or digestible energy measured in actual experiments.

The capacity of ruminants to digest high-fiber forages is significantly greater than that of pigs and chickens due to substantial differences in digestive physiology between ruminants and monogastric animals (26). *R. acetosa* L. is utilized as fodder for small ruminants and contains 12.19% CP and 8.35% digestible protein, which constitutes 67.67% of the CP. This percentage is lower than the 77.3% found in alfalfa (Table 1). Its digestible energy for ruminants is 10.11 MJ/kg, which is comparable to alfalfa’s 10.81 MJ/kg (27). Therefore, *Rumex* K-1 and Edible Grass (*R. P* × *R. T*.) present greater developmental potential as high-protein ruminant forages from the perspective of feed ingredients. The CP level of premium high-protein *Rumex* cultivars is 8–12% higher than that of alfalfa, enabling a reduction in the inclusion levels of protein feeds such as cottonseed meal and soybean meal in concentrate formulations while serving as an alternative to alfalfa. The experimental findings of Hu et al. (28) support this conclusion.

According to extensive research, the ash content of *Rumex* species and Edible Grass (*R. P* × *R. T*.) exceeds 10%, surpassing that of maize (1.4%), soybean meal (6.1%), and alfalfa (7.6%) (Table 1). Ash, defined as the mineral residue remaining after the combustion of a plant sample at high temperatures, serves as a critical indicator of the sample’s overall mineral content, encompassing both macro and trace elements (29). While leafy plants such as alfalfa, Edible Grass (*R. P* × *R. T*.), and *Rumex* exhibit significantly higher ash contents (8–12%), corn grain, functioning as a storage organ, possesses a comparatively low ash content (approximately 1.4%). The composition of the cell wall and fiber content indirectly influences this discrepancy. Leafy plants require enhanced mechanical support, leading to the accumulation of cellulose and hemicellulose-rich cell walls that absorb additional minerals, such as calcium and magnesium, thereby increasing the ash load. As cellulose deposition progresses, variations in the amount of DM occur, resulting in lower ash content during the vegetative growth stage compared to maturity. Higher dietary fiber is often correlated with increased ash content, which may negatively affect nutritional digestibility (30, 31). Furthermore, plant variety and growth environment significantly influence ash content. For example, halophytes such as *Suaeda fruticosa* and *Alhagi maurorum*, adapted to high-salinity conditions, exhibit ash contents ranging from 7.9 to 33.2%, considerably higher than typical forages (32). Breeding efforts can optimize mineral accumulation, as evidenced by the variation in ash content among genotypes of small burnet *Sanguisorba minor*, which ranges from 5.56 to 14.41% (33).

In conclusion, high-protein *Rumex* species, such as Edible Grass (*R. P × R. T.*) and *Rumex* K-1, provide significant benefits to the forage industry due to their elevated protein content. However, their widespread application in monogastric animal diets is limited by uncertainties in determining their energy value. Conversely, ruminants exhibit greater developmental potential owing to their digestive physiology, which enables them to utilize these high-fiber forages more efficiently.

### 3.2 Amino acid composition and nutritional quality evaluation

*Rumex* K-1 and Edible Grass (*R. P. × R. T.*) contain over 17 distinct types of amino acids, all of which play a vital role in human health (22). A fundamental technique for evaluating the quality of a single protein source involves scoring its AA content, a method that has progressed from rudimentary to sophisticated. One basic approach is the Amino Acid Score (AAS), which is calculated by comparing the concentration of the first limiting AA in a dietary protein to that of a reference protein, such as egg protein (34). Another widely used measure, the Protein Digestibility-Corrected Amino Acid Score (PDCAAS), incorporates protein digestibility into the AAS framework (35) Currently, the Digestible Indispensable Amino Acid Score (DIAAS) is recognized as the most accurate method for assessing the quality of a single protein source, as it utilizes true ileal amino acid digestibility, providing a more precise reflection of AA absorption in the human small intestine (36).

The Digestible Indispensable Amino Acid Score (DIAAS) provides a more accurate representation of protein utilization in the human body compared to the conventional Protein Digestibility-Corrected Amino Acid Score (PDCAAS). The Food and Agriculture Organization of the United Nations (FAO) has recommended DIAAS for assessing protein quality in human foods. Currently, there are no published studies that employ both PDCAAS and DIAAS techniques to evaluate high-protein *Rumex* variants. This study aims to estimate the protein value of various forage crops by thoroughly matching the AA values reported in the literature with the optimal protein pattern suggested by the FAO/WHO (Table 2). For the essential amino acids—Threonine (Thr), Valine (Val), Methionine + Cysteine (Met+Cys), Isoleucine (Ile), Leucine (Leu), Phenylalanine (Phe), and Lysine (Lys)—the amino acid scores (AAS) were as follows: soybean meal > Edible Grass (*R. P* × *R. T*.) > alfalfa > *Rumex* K-1. This indicates that soybean meal has a significant advantage in terms of protein quality, while the other forage crops exhibit comparatively lower values. Forage crops such as alfalfa (37), *Rumex* K-1 (38), and Edible Grass (*R. P* × *R. T*.) (39) are characterized by relatively low methionine content in their AA composition, which may significantly restrict their potential as high-quality protein sources. Methionine serves as the first limiting AA in poultry diets and the second limiting AA in pig diets, and its deficiency can adversely affect animal growth performance and feed efficiency. Therefore, optimizing or supplementing methionine in feed formulation should be given particular attention.

**Table 2:** Amino acid score of *Rumex* plants.

Species	CP, %	Thr	Val	Met+Cys	Ile	Leu	Phe	Lys	Ref.
Soybean meal	44.0	0.539	0.447	0.417	0.506	0.523	0.418	0.496	(37)
Alfalfa	19.0	0.213	0.209	0.141	0.195	0.197	0.157	0.171	(37)
Edible Grass (*R. P. × R. T.*)	30.15	0.331	0.323	0.302	0.326	0.326	0.232	0.188	(39)
*Rumex* K-1	19.24	0.178	0.154	0.131	0.168	0.295	0.122	0.193	(38)
*R. acetosa* L.	25.00	0.350	0.390	0.382	0.233	0.343	0.115	0.250	(70)
*R. crispus*	-	0.2500	0.1978	-	0.1885	0.141	0.148	0.103	(74)

The ideal protein pattern recommended by FAO/WHO: Threonine: 4.0%, Valine: 5.0%, Methionine + Cystine: 3.5%, Isoleucine: 4.0%, Leucine: 7.0%, Phenylalanine: 6.0%, Lysine: 5.5%. Amino acid score (AAS) = amino acid content in the sample protein / content of the corresponding essential amino acids in the FAO/WHO scoring standard model. The calculation of AAS is based on the DM basis.

### 3.3 Bioactive components

Several species of *Rumex* have a long history of use as medicinal plants. Recent metabolic analyses of nearly 29 *Rumex* species have identified approximately 268 chemical compounds, predominantly consisting of anthraquinones, phenolic acids, flavonoids, tannins, and diterpene alkaloids (10). This review focuses on the bioactive components present in forage varieties, specifically *R. acetosa* L., *Rumex* K-1, and Edible Grass (*R. P* × *R. T*.). These components have the potential to enhance livestock production performance and improve health status through their anti-inflammatory, antioxidant, and immune-enhancing effects (5, 13, 22). Research by Omarova et al. (15) on *Rumex* K-1 at the early flowering stage showed that the anthracene derivative content was 350 mg/100 g in leaves and 110 mg/100 g in roots. The total phenolic content was 10.2% in leaves and 3.4% in roots. The flavonoid content was 5.1% in leaves and 0.7% in roots. Khodzhaeva et al. (16) determined the flavonoid content in *Rumex* K-1 to be 0.17% using a method involving extraction with 70% aqueous ethanol, concentration, and CHCl₃ extraction. Hu et al. (39) investigated the flavonoid and phenolic acid contents in the leaves, roots, stems, and seeds of Edible Grass (*R. P* × *R. T*.) during its growing stage. The seeds exhibited the highest flavonoid content at 0.45%, followed by the roots at 0.35% and the leaves at 0.13%. In contrast, the leaves contained the highest phenolic acid content at 1.98%, with the roots following at 1.74%. Tannin content was relatively consistent across all four plant parts, averaging approximately 0.25%.

## 4 Main anti-nutritional factors (ANFs)

### 4.1 Types of ANFs

Plant-based foods are a significant source of nutrition; however, their nutritional value is not solely determined by the total nutrient content. The critical aspect lies in the bioavailability of these nutrients. A variety of natural compounds, referred to as ANFs, can substantially interfere with this process by hindering the digestion, absorption, and utilization of essential nutrients through various mechanisms (40). Oxalic acid and phytic acid are naturally occurring organic acids commonly found in many plants. Vegetables that are high in oxalic acid include spinach and purslane, among others. Phytic acid typically exists in the form of phytate phosphorus and is predominantly found in seeds. Oxalic acid is frequently present in the roots and leaves of plants such as rhubarb, tea, spinach, parsley, purslane, and beet (41). The primary anti-nutritional mechanism involves the formation of insoluble salts, such as calcium oxalate, with minerals, particularly calcium and iron. These precipitates cannot be absorbed by the intestine, leading directly to reduced mineral bioavailability and potentially causing hyperoxaluria, which may subsequently result in kidney stones (42). The formation of insoluble salts, such as calcium oxalate, with minerals—particularly calcium and iron—represents a primary anti-nutritional mechanism. The intestine is unable to absorb these precipitates, which significantly reduces mineral bioavailability and may lead to hyperoxaluria, subsequently resulting in kidney stones. The principal anti-nutritional component found in plants of the genus *Rumex* is oxalic acid. Numerous studies have confirmed the high concentration of oxalic acid in various *Rumex* species. For instance, oxalic acid has been detected in the roots and leaves of *R. crispus* (43, 44). The oxalate metabolism of *R. obtusifolius* has been the focus of specific research (45). A report detailing a sheep poisoning incident indicated that the oxalic acid levels in the DM of *R. crispus* responsible for the poisoning ranged from 6.6 to 11.1% (46). Additionally, the leaves of *R. induratu*s have been shown to contain elevated levels (5.17%) of oxalic acid (47). In a study of wild edible plants in Ethiopia, *R. nervosus* was also found to contain oxalic acid (48). Furthermore, the leaves of *R. acetosa* L. have been identified to possess hazardous amounts of oxalic acid (49). These findings suggest that many *Rumex* species exhibit high concentrations of oxalic acid. The DM of Edible Grass (*R. P. × R. T.*) contained 0.6% phytic acid and 10.3% oxalic acid in the leaves, while the roots contained 7.2% (39). The leaves and roots of *R. crispus* had phytic acid concentrations of 1.15 ± 0.74% and 1.38 ± 0.27%, respectively (43). The leaves of *R. acetosa* had phytic acid concentrations of 0.31–0.62% (50). Table 3 summarizes the main anti-nutritional factors in different *Rumex* species.

**Table 3:** Main anti-nutritional factors in *Rumex* plants.

Species	Plant parts	State	Anti-nutrition factors content, %			Ref.
			Oxalic acid	Phytic acid	Tannin	
Edible Grass (R. P. *×*R. T.)	Leaf	Air-dried	10.32	6.75	0.25	(39)
	Root		7.21	4.64	0.27	
	Stem		6.30	4.85	0.25	
	Seed		4.96	2.75	0.28	
	Leaf & stem	Air-dried	0.32	—	—	(75)
*R.* OK2	Whole-plant	DM	3–13	—	—	(6)
*R. crispus*	Leaf	DM	6.6–11	—	—	(46)
	Leaf	DM	0.015	1.15	—	(43)
	Root	DM	0.011	1.38	—	
*R. acetosa*	Leaf	DM	—	—	—	(50)
*R. obtusifolius*	Leaf	Fresh	0.45	—	—	(45)
*R. Induratus*	Leaf	DM	5.17	—	—	(47)
*R. nervosus*	Young shoot	Fresh	0.005	—	0.002	(48)

“Whole-plant” is widely used in silage. Generally, it refers to the entire above - ground part of the plant at a certain growth stage, including stems, leaves and fruits.

NSP represent a significant class of ANFs in plant-based feed ingredients for monogastric animals. NSP primarily encompasses carbohydrate components such as cellulose, hemicellulose, and pectin, which are not hydrolyzed by the endogenous digestive enzymes of monogastric animals. NSP exhibits two principal types of anti-nutritional effects: biochemical and physical. In the intestinal tract, soluble NSP (including arabinoxylan and *β*-glucan) can form highly viscous gel-like solutions. This viscous environment diminishes the efficacy of nutrient digestion and absorption by hindering the interaction between digestive enzymes and substrates, while also slowing the transit velocity of digesta. Biochemically, the structural characteristics of NSP may lead to their binding with endogenous substances (such as bile acids and digestive enzymes) or serve as substrates for excessive fermentation by intestinal microorganisms, thereby disrupting the equilibrium of gut microbiota and producing adverse effects (45). A study conducted by Li et al. (24) indicated that the inclusion of 12% Edible Grass (*R. P* × *R. T*.) in the diet resulted in a decline in broiler developmental performance, likely due to the excessively high levels of oxalic acid and NSP in the diet. Moreover, current research on NSP in *Rumex* plants has primarily focused on structural fiber components (as reflected in CF, NDF and ADF values in Table 1), while the composition and anti-nutritional effects of soluble NSP (e.g., *β*-glucan, arabinoxylan) in monogastric animals have not yet been systematically reported. Given that soluble NSP are key factors affecting dietary digestibility and gut health, future research should prioritize quantitative analysis of soluble NSP in *Rumex* and evaluate their impact on digestive physiology in livestock and poultry, in order to establish safe inclusion thresholds in high-proportion diets.

### 4.2 Measures to reduce or eliminate ANFs

At present, there have been relevant reports on papers regarding the reduction of oxalic acid content in *Rumex* Plants by microbial methods. Fermentation has been widely regarded as a bioprocessing approach capable of enhancing the flavor characteristics and nutritional value of food ingredients (51). Fermentation can effectively reduce the content of anti - nutritional factors, thus positively influencing the health benefits and nutritional quality of the final product (52). The research by Li et al. (53) shows that the relative abundance of oxalic acid in Edible Grass (*R. P. × R. T.*) after 124 days of fermentation is significantly lower than that after 17 days of fermentation. Further research by Li et al. (54) indicates that both single fermentation and combined fermentation using *Lactobacillus plantarum* and *Lactobacillus rhamnosus* can significantly reduce the oxalic acid content in Edible Grass (*R. P × R. T.*). The combined fermentation shows the best effect, with the oxalic acid content decreasing by approximately 75.98% after 7 days of fermentation (2.423 mg/mL before fermentation vs. 0.258 mg/mL after fermentation). Research by Rolinec et al. (6) shows that silage can also reduce the oxalic acid content in *Rumex* OK2.

## 5 Research progress on the application in livestock and poultry production

### 5.1 Application in ruminants

Ensiling and fermenting are critical technological processes that enhance the storability of Edible Grass (*R. P × R. T.*), particularly because its moisture content at the leaf-cluster stage exceeds 90% (Table 1). The incorporation of lactic acid bacteria significantly improves both the fermentation quality and nutritional value of Edible Grass (*R. P × R. T.*) silage at a moisture content of 60%. A study by Zhou et al. (55) indicated that the DM disappearance rate, assessed using sheep rumen fluid, was markedly increased following this treatment. Furthermore, Chen et al. (56) found that the silage quality of high-moisture Edible Grass (R. P × R. T.) (moisture content >90%) could be substantially improved by adjusting wilting time and employing additives. Rumen degradation tests, utilizing rumen fluid from Small-tailed Han sheep, revealed that the 48-h degradation rates of DM and CP in Edible Grass (*R. P. × R. T.*) were 81.05 and 82.88%, respectively, with effective degradation rates of 56.13 and 58.93%, indicating favorable degradation characteristics in the rumen of meat sheep (57). Additionally, research by Xu et al. (58) demonstrated that Edible Grass (*R. P. × R. T.*) harvested at 25 days of growth exhibited optimal rumen degradation characteristics and the highest nutritional value, with rumen DM degradation rates of at least 71.79% and CP degradation rates of at least 77.78% during this growth period. Hu (28) determined that incorporating 12% ensiled Edible Grass (*R. P. × R. T.*) into black goat diets, as assessed by an *in vitro* gas generation method utilizing rumen fluid from black goats, resulted in the highest total volatile fatty acid concentration in the rumen. The effective degradation rates of NDF, DM, and CP were comparable to those of the control group, which consisted of 9.3% soybean meal and 24% alfalfa, indicating that fiber degradation remained unaffected. Subsequent *in vivo* animal trials revealed that substituting 12% ensiled Edible Grass (*R. P. × R. T.*) for 12% alfalfa and 3.98% soybean meal did not significantly impact the growth performance, nutrient apparent digestibility, rumen fermentation characteristics, slaughter performance, serum biochemical parameters, or antioxidant indices of black goats, although there was a slight improvement in meat quality. In addition to enhancing growth performance, slaughter performance, nutrient apparent digestibility, and meat quality, Li et al. (59) found that supplementing sheep diets with 5% or 10% fermented Edible Grass (*R. P. × R. T.*) in place of an equivalent amount of soybean meal significantly improved markers of blood protein metabolism. Comprehensive data indicated that the 5% replacement group outperformed the others, effectively promoting fat deposition and protein metabolism. The relative abundance of *Butyrivibrio* was significantly higher in the 10% fermented Edible Grass (*R. P. × R. T.*) group, which also exhibited markedly greater rumen microbial richness and diversity. Although the dominant phyla, such as *Firmicutes* and *Bacteroidota*, did not undergo substantial changes, the adjustment of the microbial community structure contributed to the maintenance of gastrointestinal health (60).

### 5.2 Application in swine production

The addition of 8–10% fresh Edible Grass (*R. P. × R. T.*) to the diet can increase the average daily gain of finishing pigs by approximately 15%, reduce the feed-to-gain ratio by about 8%, improve carcass quality, increase serum total protein and globulin levels, and decrease blood urea nitrogen content. These findings indicate a reduced metabolic burden on the liver and improved protein utilization (61). Lei et al. (25) showed that dietary supplementation with a total mixed ration of fermented Edible Grass (*R. P. × R. T.*) at varying protein levels (10.30–14.13%) significantly enhanced the growth performance and carcass quality of finishing pigs. Compared with the control group (16.11% protein), the average daily gain in all three treatment groups was significantly increased. Among these, Treatment III (10.30% protein) achieved the lowest feed-to-gain ratio, the best feed conversion efficiency, and a significantly higher lean meat percentage. Increasing the inclusion level of *Rumex* K-1 meal in the diet (from 0 to 6%) linearly reduced DM digestibility and calcium utilization, and decreased the average daily gain of growing pigs during the first two weeks. However, the addition of 0.15% cellulase to the diet containing 6% *Rumex* K-1 meal significantly improved the digestibility of DM, organic matter, gross energy, and acid detergent fiber, and enhanced the average daily gain during the initial two-week period (24).

### 5.3 Application in poultry production

Recent studies have highlighted the utilization of *Rumex* plant resources in the poultry industry. Li et al. (22) found that substituting 3–6% Edible Grass (*R. P. × R. T.*) meal for soybean meal in the diets of AA white feather broilers resulted in improved growth performance, enhanced antioxidant capacity, increased cecal short-chain fatty acid content, and a higher presence of beneficial bacteria such as *Lactobacillus*. However, it was observed that broiler growth performance declined when the inclusion level reached 12.0%. Additionally, Nong et al. (62) reported that feeding 5.0% fermented Edible Grass (*R. P. × R. T.*) to local meat chickens (Lingshan native chickens) led to improved villus morphology in the jejunum and ileum, a reduced feed-to-gain ratio, and an optimized cecal microbiota characterized by an increased abundance of *Verrucomicrobiota* and the genus *WCHB1-41*. These findings indicate that fermented Edible Grass (*R. P. × R. T.*) effectively promotes intestinal health and enhances growth performance in broilers.

To enhance broiler growth performance, immune function, antioxidant capacity, and intestinal health, Banday et al. (13) demonstrated that incorporating 0.5–1.0% *R. nepalensis* leaf powder into the diet significantly increased weight gain and feed conversion rates, reduced serum total cholesterol, elevated immunoglobulin levels and antioxidant enzyme activities, optimized cecal microbiota (resulting in decreased *Escherichia coli* counts), and improved the villus structure of both the duodenum and jejunum. Similarly, Qaid et al. (63) reported that supplementing Ross broilers’ diets with 0.1–0.5% *R. nervosus* leaf powder enhanced intestinal growth, carcass characteristics, and economic efficiency. Notably, a supplementation level of 0.3% was found to be optimal for the ileum and the entire small intestine, while 0.5% supplementation significantly increased both the weight and thickness of the duodenum and jejunum. Additionally, the 0.3% supplementation notably improved carcass weight and dressing percentage. From an economic perspective, the low-dose group (0.1%) exhibited the lowest cost–benefit ratio, yet it generated the highest revenue and net profit. Furthermore, broilers infected with Eimeria experienced an anticoccidial effect when fed 0.1–0.5% *R. nervosus* leaf powder. Supplementation with 0.1–0.5% *R. nervosus* leaf powder led to a significant reduction in intestinal lesion scores and fecal oocyst shedding compared to the control group. Specifically, the 0.1% supplementation group markedly improved the feed conversion rate in infected broilers, while the 0.5% supplementation group provided mild anticoccidial benefits. These findings suggest that *R. nervosus* leaf powder, at a recommended dosage of 0.1–0.5%, may serve as an effective adjuvant treatment for avian coccidiosis (64).

Research by Wang et al. (5) demonstrates that *Rumex* K-1 leaf powder effectively promotes growth and intestinal health in geese. Their study found that incorporating 1.0 to 4.0% of the powder into the diet significantly improved feed conversion efficiency in Sanhua geese, enhanced the development of the liver and proventriculus, reduced serum AST and GLU levels, and optimized the structure of the cecal microbiota by enriching beneficial bacterial communities. Furthermore, subsequent studies indicated that the addition of 2.5% *Rumex* K-1 leaf powder to a low-protein diet further enhanced the cecal microbiota, growth performance, and slaughter characteristics in Sanhua geese (23).

## 6 Obstacles and prospects for the future

Based on the preceding discussion, we posit that the bioavailability of certain minerals, particularly calcium, is diminished in high-protein *Rumex* variants due to elevated levels of the anti-nutritional factor, oxalic acid. A significant barrier to its utilization as a food ingredient, especially as a feed component, is the resultant mineral deficiency (65). One effective approach to mitigate ANFs is through fermentation. During this process, microorganisms secrete enzymes such as phytase, tannase, and proteases that can hydrolyze these compounds. Concurrently, microbial metabolism generates organic acids (e.g., lactic acid) that lower pH levels, thereby activating endogenous enzymes and altering substrate solubility, which aids in the degradation of anti-nutritional substances (66). Although pigs and ruminants have been fed fermented and ensiled Edible Grass (*R. P* × *R. T*.), there remains a paucity of information regarding their oxalic acid content, highlighting the need for further research in this domain. Another strategy for diminishing oxalic acid and NSP in the raw material is protein extraction. The levels of oxalic acid and NSP can be significantly reduced by pulping fresh Edible Grass (*R. P* × *R. T*.) leaves and employing physicochemical methods for protein extraction. This technique can decrease oxalic acid concentration to 2.7% and CF content to below 5% (unpublished data). Current research in this field faces several challenges, including a lack of quantified energy levels, considerable variability in nutrient composition across different varieties and growth stages, and unclear mechanisms of action for bioactive components. To foster the scientific application and industrial development of *Rumex* plants as high-quality protein feed resources, future efforts should focus on varietal improvement, standardization of processing technologies, foundational studies on energy metabolism, and mechanistic investigations. In particular, soluble NSP (e.g., *β*-glucan) in *Rumex* remain uncharacterized. Future studies should quantify them, assess their anti-nutritional effects, and develop enzyme strategies to enable higher inclusion levels in monogastric diets.

The modern feed industry demands high stability in the nutritional composition of feed ingredients. Table 1 shows that the nutritional composition of high-protein *Rumex* varieties varies significantly across different varieties and growth stages, with a relatively low level of standardization. The CP content of Edible Grass (*R. P. × R. T.*) and *Rumex* K-1 fluctuates between 19 and 30%. The coefficients of variation for key nutritional indicators are considerable, and lower protein content is often accompanied by higher fiber content. According to literature reports, the optimal harvest period for high-protein *Rumex* varieties such as Edible Grass (*R. P. × R. T.*) and *Rumex* K-1 is the leaf-cluster stage of the growth period (67). Inconsistent harvesting times may be an important cause of nutritional indicator fluctuations. Furthermore, extensive commercial promotion has exaggerated the stress resistance of some varieties, lacking sufficient support from field trial data. For example, extrapolating annual yield and protein yield based solely on yields from a single cutting or two to three cuttings overlooks the actual growth performance of the varieties under different climatic conditions. Recent studies indicate that high-temperature environments in subtropical regions can lead to reduced yield or even death of Edible Grass (*R. P. × R. T.*), while agricultural measures such as shading can effectively alleviate heat stress (67). Currently, research on the effects of environmental factors such as temperature, humidity, and light on the physiological characteristics and nutritional indicators of high-protein *Rumex* varieties remains insufficient, with a lack of adequate supporting data, further exacerbating the instability of processed products.

Currently, a total of 28 *Rumex* resources are listed in Table 4, most of which are wild varieties. Systematically screening and exploring germplasm with desirable traits, such as disease resistance, drought tolerance, and high yield, from these abundant wild resources represents an important direction for the future development and utilization of *Rumex* plant resources. Utilizing modern biotechnological tools such as cross-breeding and gene editing to introduce desirable traits from wild varieties into cultivated varieties holds promise for developing new varieties with broader adaptability, more stable nutritional composition, and enhanced stress resistance (68). Concurrently, conducting regional variety adaptability trials tailored to the climatic conditions of different areas, and establishing standardized cultivation technical specifications encompassing optimal harvesting periods, planting densities, and water and fertilizer management practices, is of great significance for improving the yield and quality stability of *Rumex* plants.

**Table 4:** Species and distribution of the genus *Rumex* L. in China.

No.	Common name/commercial name	Scientific name (in Latin)	Recommended inclusion level (%)	Distribution	Application
R1	Sorrel, dock, sour weed	*R. acetosa* L.	*—*	East Asia (including both northern and southern China), the Caucasus, Kazakhstan, Russia, Europe, and the Americas	Edible/feed/medicinal (14, 65)
R2	—	*R. acetosella* L.	*—*	Heilongjiang, Inner Mongolia, Xinjiang, Hebei, Shandong, Henan, Jiangxi, Hunan, Hubei, Sichuan, Fujian, and Taiwan; Korea, Japan, Mongolia, the Caucasus, Kazakhstan, Russia, Europe, and North America.	—
R3	Yangtie leaf, Yangti sorrel	*R. japonicus* Houtt	*—*	Northeast China, North China, East China, Central China, South China, Shaanxi, Sichuan and Guizhou; North Korea, Japan, and Russia (Far East).	Medicinal (14, 76)
R4	Patienta dock	*R. patientia L.*	*—*	Northeast China, North China, Northwest China, Shandong, Henan, Hunan, Hubei, Sichuan, and Xizang (Tibet); the Caucasus, Kazakhstan, Russia, Mongolia, and Europe.	Medicinal (14)
R5	Tudahuang	*R. crispus* L.	*—*	Northeast China, North China, Northwest China, Shandong, Henan, Hubei, Sichuan, Guizhou, and Yunnan; the Caucasus, Kazakhstan, Russia (Siberia, Russian Far East), Mongolia, Korea, Japan, Europe, and North America.	Medicinal (12, 14, 77)
R6	—	*R. obtusifolius L.*	*—*	Hebei, Shandong, Shaanxi, Gansu, Jiangsu, Zhejiang, Jiangxi, Anhui, Hunan, Hubei, and Sichuan; Japan, Europe, and Africa.	Medicinal (14)
R7	—	*R. trisetifer* Stokes	*—*	Shaanxi, Jiangsu, Zhejiang, Anhui, Jiangxi, Hunan, Hubei, Sichuan, Taiwan, Fujian, Guangdong, Hainan, Guangxi, Guizhou, and Yunnan; Vietnam, Laos, Thailand, Bangladesh, and India.	Medicinal (12, 76, 77)
R8	—	*R. longifolius* DC.	*—*	Northeast China, North China, Northwest China, Shandong, Henan, Hubei, and Sichuan; Japan, Russia, Europe, and North America.	—
R9	—	*R. nepalensis*	Broiler Chicken: 0.5–1%	Shaanxi, Gansu, Qinghai, Hunan, Hubei, Jiangxi, Sichuan, Guangxi, Guizhou, Yunnan, and Tibet; Iran, Afghanistan, India, Pakistan, Nepal, Myanmar, Vietnam, and Indonesia.	Edible/feed/medicinal (13, 78)
R10	—	*R. dentatus L.*	*—*	North China, Northwest China, East China, Central China, Sichuan, Guizhou, and Yunnan; Nepal, India, Afghanistan, Kazakhstan, and Southeastern Europe.	Medicinal
R11	—	*R. maritimus L.*	*—*	Northeast China, North China, Northern Shaanxi, and Xinjiang; The Caucasus, Kazakhstan, Russia (Siberia, Russian Far East), Mongolia, Europe, and North America.	—
R12	—	*R.marschallianus Rchb.*	*—*	Inner Mongolia and Xinjiang; Mongolia, Russia (Siberia, the southeastern part of its European territory), Kazakhstan, and Ukraine.	—
R13	—	*R. amurensis F. Schmidt ex Maxim.*	*—*	Northeast China, Hebei, Henan, Shandong, Jiangsu, and Anhui; Distributed in Russia (Russian Far East).	—
R14	Hongsi sorrel Niuxixi	*R. chalepensis Mill.*	*—*	Hebei, Shanxi, Shandong, Henan, Hubei, Shaanxi, Gansu, Xinjiang, Jiangsu, Zhejiang, and Anhui; Iraq, Iran, Afghanistan, Pakistan, Kyrgyzstan, and the Kashmir region.	—
R15	—	*R. gmelinii Turcz. ex Ledeb.*	*—*	China, North China, Shaanxi, Gansu, Qinghai (Menyuan), and Xinjiang (Altay); Korea, Japan, Mongolia, and Russia (Siberia, Russian Far East). Altitude: 300–1,200 meters.	—
R16	—	*R. hastatus D. Don*	*—*	Yunnan, Sichuan, and Southeastern Tibet; India, Nepal, Bhutan, Pakistan, and Afghanistan.	—
R17	—	*R. angulatus Rech. F.*	*—*	Tibet; Pakistan, the Kashmir region, and Afghanistan.	—
R18	—	*R. aquaticus L.*	*—*	Heilongjiang, Jilin, Shanxi, Shaanxi, Ningxia, Gansu, Qinghai, Xinjiang, Western Hubei, and Sichuan (Mao’ergai); Japan, Mongolia, the Caucasus, Kazakhstan, Russia, and Europe.	—
R19	—	*R. microcarpus Campd.*	*—*	Liaoning, Hebei, Jiangsu, Taiwan, Hainan, Guangxi, Guizhou, and Yunnan, among others; Bangladesh, Vietnam, and India.	—
R20	—	*R. popovii Pachom.*	*—*	Xinjiang; Mongolia, Tajikistan, and Kazakhstan.	—
R21	—	*R. tianshanicus A. Los*	*—*	Xinjiang; Kazakhstan.	—
R22	—	*R. pseudonatronatus (Borbás) Borbás ex Murb.*	*—*	Heilongjiang, Jilin, Hebei, Shaanxi, Gansu, Qinghai, and Xinjiang; Mongolia, the Caucasus, Kazakhstan, Russia, and Europe.	—
R23	—	*R. stenophyllus Ledeb.*	*—*	Heilongjiang (Huachuan), Jilin (Hunchun), Inner Mongolia, and Xinjiang; Mongolia, the Caucasus, Kazakhstan, Russia, and Europe.	—
R24	—	*R. thyrsiflorus Fingerh.*	*—*	Heilongjiang (Huachuan), Jilin (Hunchun), Inner Mongolia, and Xinjiang; Kazakhstan, Russia, and Europe.	—
R25	—	*R. ucranicus Fisch. ex Spreng.*	*—*	Xinjiang; Poland, Ukraine, Kazakhstan, and Russia (European part).	—
R26	—	*R. yungningensis Sam.*	*—*	Northwestern Yunnan and Southwestern Sichuan.	—
R27	Edible Dock *Rumex* K-1	*R. patientia L. × R. tianschanicus* A. LOS	Goslings: 1–2.5%; Pig: 3–6%;	Northeast China, North China, Northwest China.	Feed (5, 24)
R28	Edible Grass (*R. P* × *R. T*.)	*R. patientia L. × R. tianschanicus* A. LOS	Broiler Chicken: 3–6%; Goat: 6–12%; Sheep: 5–10%	Northeast China, North China, Northwest China, Shandong, Henan, Hubei, Hunan and Sichuan;	Edible/feed (22, 28, 59)

The names, Latin names, and distribution are sourced from Flora of China. The “Recommended inclusion level (%)” is either a value that, as reported in the literature, has no negative impact on the growth performance and health of animals, or a recommended value provided by the authors of the paper.

## 7 Conclusion

**Figure 3:**
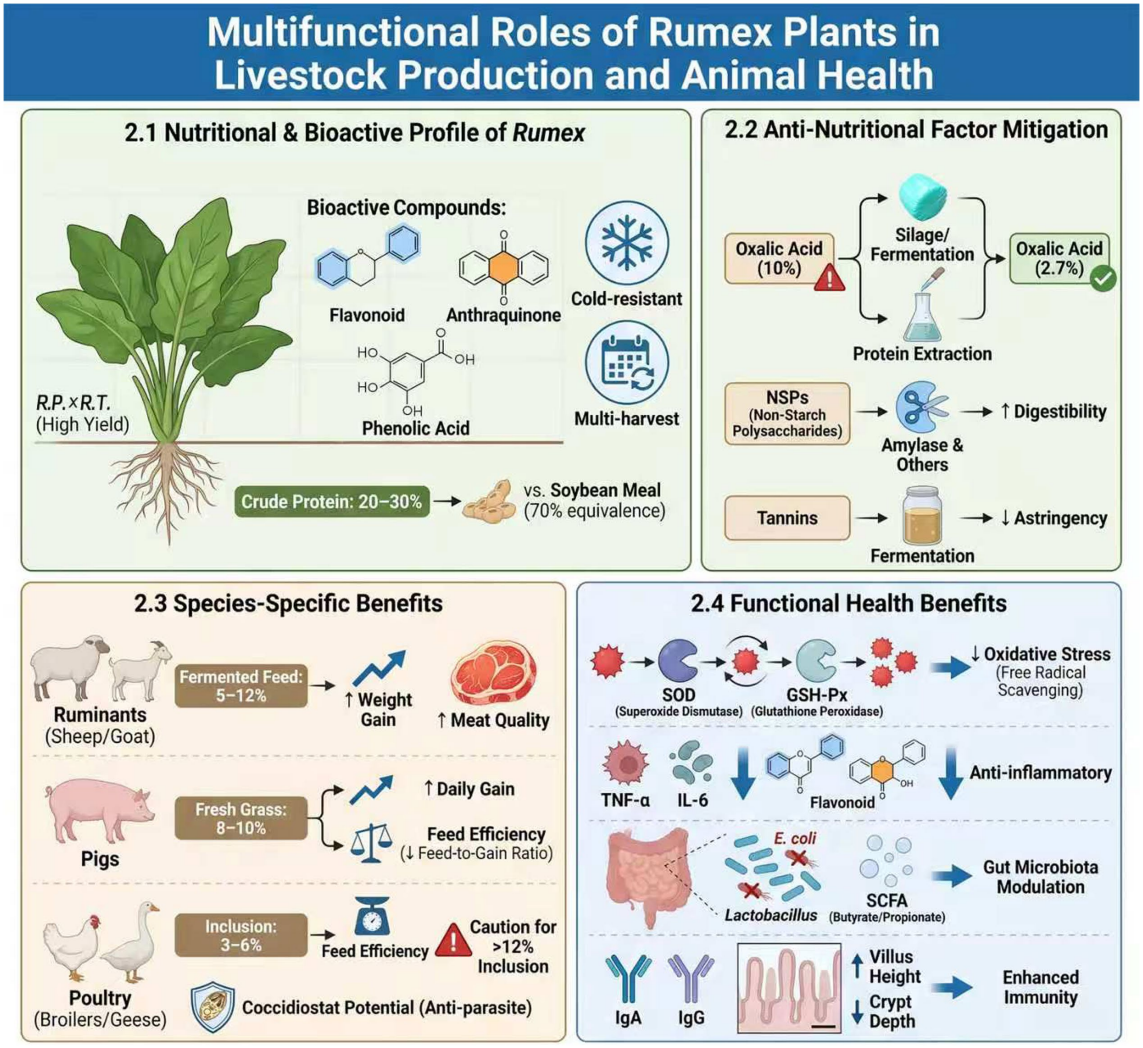
Multifunctional roles of *Rumex* plants in livestock production and animal health.

*Rumex* plants, such as Edible Grass (*R. P. × R. T.*) and *Rumex* K-1, exhibit significant potential as novel protein feed resources due to their high protein content of 20–30%, high yield, and abundance of bioactive components including anthraquinones and flavonoids. However, their application and promotion face dual challenges: on one hand, the presence of ANFs such as oxalic acid and NSP limits their inclusion levels in diets; on the other hand, the lack of measured energy values and imbalances in AA composition, particularly methionine deficiency, constrain their use in precision diets for monogastric animals. Current research indicates that selecting appropriate harvest stages and employing processing methods such as ensiling and fermentation can effectively reduce the content of ANFs. Animal trials have confirmed that moderate inclusion of fermented *Rumex* products in the diets of ruminants at 5–12% and monogastric animals at 3–10% can improve growth performance, antioxidant capacity, and intestinal health. However, inclusion levels exceeding the threshold, such as 12% in poultry, can lead to negative effects, highlighting the need for future research to establish safe inclusion ranges for different animal species. In Figure 3, we provide a visual description of “Multifunctional Roles of *Rumex* Plants in Livestock Production and Animal Health”. In summary, fully realizing the feed value of *Rumex* plants requires systematic advancements in variety selection, hazard reduction processing, precise evaluation, and application validation. Future efforts should focus on the following areas: (1) breeding specialized varieties with low oxalic acid and high protein content; (2) accurately assessing protein quality using methods such as DIAAS and determining true energy values through the net energy system; (3) employing omics technologies to elucidate the molecular mechanisms by which bioactive components such as flavonoids and anthraquinones regulate intestinal health and antioxidant function; and (4) conducting long-term, large-sample animal trials to establish optimal application strategies under antibiotic-free farming conditions.

## Glossary

ANFs: anti-nutritional factors
NSP: non-starch polysaccharides
*R. P. × R. T.*: *Rumex patientia* L. × *Rumex tianschanicus* A. LOS
*R.*: *Rumex*
DM: dry matter
CP: crude protein
EE: ether extract
Ash: crude ash
CF: crude fiber
NFE: nitrogen-free extract
ME: metabolizable energy
DE: digestible energy
BW: body weight
NDF: neutral detergent fiber
ADF: acid detergent fiber
Thr: Threonine
Val: Valine
Met: Methionine
Cys: Cystine
Ile: Isoleucine
Leu: Leucine
Phe: Phenylalanine
Lys: Lysine
AA: Amino Acid
AAS: Amino Acid Score
PDCAAS: Protein Digestibility-Corrected Amino Acid Score
DIAAS: Digestible Indispensable Amino Acid Score
AST: Aspartate Aminotransferase
GLU: Glucose
